# Food Insecurity in Pregnancy, Receipt of Food Assistance, and Perinatal Complications

**DOI:** 10.1001/jamanetworkopen.2024.55955

**Published:** 2025-01-23

**Authors:** Rana F. Chehab, Lisa A. Croen, Barbara A. Laraia, Mara B. Greenberg, Amanda L. Ngo, Assiamira Ferrara, Yeyi Zhu

**Affiliations:** 1 Division of Research, Kaiser Permanente Northern California, Pleasanton; 2 Center for Upstream Prevention of Adiposity and Diabetes Mellitus, Pleasanton, California; 3 Department of Obstetrics and Gynecology, Kaiser Permanente Northern California, Oakland; 4 Regional Perinatal Service Center, Kaiser Permanente Northern California, Santa Clara; 5 School of Public Health, University of California, Berkeley; 6 Department of Epidemiology and Biostatistics, University of California, San Francisco

## Abstract

**Importance:**

Food insecurity is a growing public health concern, but its association with perinatal complications remains unclear.

**Objective:**

To examine whether food insecurity in pregnancy was associated with the risk of perinatal complications and determine whether these potential associations differed by receipt of food assistance.

**Design, Setting, and Participants:**

This cohort study used data from a pregnancy survey conducted between June 22, 2020, and September 9, 2022, at Kaiser Permanente Northern California, an integrated health care system serving a diverse population of 4.6 million. Participants included individuals who delivered singletons. Data were analyzed from December 2023 to June 2024.

**Exposure:**

Food insecurity in pregnancy assessed using the validated 2-item Hunger Vital Sign screener.

**Main Outcomes and Measures:**

Maternal (gestational diabetes, gestational hypertension, preeclampsia, cesarean delivery) and neonatal (preterm birth, neonatal intensive care unit [NICU] admission, small-for-gestational age [SGA], and large-for-gestational age [LGA]) complications extracted from the electronic health records, and a composite adverse perinatal outcome (APO) of maternal and neonatal complications. Modified Poisson regression models were adjusted for covariates and stratified by receipt of food assistance in pregnancy.

**Results:**

Among 19 338 individuals, 2707 (14.0%) reported food insecurity in pregnancy. Individuals with food insecurity in pregnancy had a higher risk of gestational diabetes (adjusted relative risk [aRR], 1.13 [95% CI, 1.01-1.29]), preeclampsia (aRR, 1.28 [95% CI, 1.11-1.49]), preterm birth (aRR, 1.19 [95% CI, 1.02-1.38]), NICU admission (aRR, 1.23 [95% CI, 1.07-1.42]), and APO (aRR, 1.07 [95% CI, 1.02-1.13]) compared with individuals without food insecurity. Among 1471 individuals (7.6%) who received food assistance in pregnancy, associations of food insecurity in pregnancy with perinatal complications were attenuated to the null, except for preeclampsia (aRR, 1.64 [95% CI, 1.06-2.53]). On the contrary, the associations persisted among individuals who did not receive food assistance: gestational diabetes (aRR, 1.20 [95% CI, 1.04-1.37]), preeclampsia (aRR, 1.24 [95% CI, 1.06-1.46]), preterm birth (aRR, 1.23 [95% CI, 1.05-1.46]), NICU admission (aRR, 1.31 [95% CI, 1.12-1.52]), and APO (aRR, 1.12 [95% CI, 1.06-1.18]).

**Conclusions and Relevance:**

In this cohort study, food insecurity in pregnancy was associated with a higher risk of perinatal complications, and these associations were overall attenuated to the null among individuals who received food assistance in pregnancy. These findings support clinical guidelines of screening for food insecurity in pregnancy and provide evidence to expand food assistance programs that may help improve maternal and neonatal outcomes.

## Key Points

**Question:**

Is food insecurity in pregnancy associated with perinatal complications and do these potential associations differ by receipt of food assistance?

**Findings:**

In this cohort study of 19 338 individuals, food insecurity in pregnancy was prevalent at 14.0% and was associated with a higher risk of gestational diabetes, preeclampsia, preterm birth, and neonatal intensive care unit admission. These associations were overall attenuated to the null among individuals who received food assistance but persisted among those who did not.

**Meaning:**

These findings suggest that the higher risk of perinatal complications associated with food insecurity in pregnancy is lar-gely attenuated among individuals who received food assistance.

## Teaser

This cohort study examines food insecurity during pregnancy among individuals in Northern California during the COVID-19 pandemic.

## Introduction

Food insecurity, a lack of consistent access to the nutritionally adequate and safe food needed for a healthy life,^1^ is a major public health problem in the US associated with adverse health sequalae and increased health care expenditure.^2,3,4^ Pregnancy is a critical period during which exposure to food insecurity can have magnified detrimental effects on the pregnant individual and their developing fetuses.^5^ National estimates of food insecurity among pregnant individuals in the US are lacking, but the available limited data among peripartum individuals indicate an alarming prevalence of 10.8% in 2019 to 2021.^6^

Albeit the growing body of evidence linking food insecurity to adverse health outcomes in the general population,^2,3,7^ less is known about this link in pregnancy. Gestational diabetes (GD) is the most commonly studied perinatal complication in terms of its association with food insecurity; however, the few existing studies are limited with a small sample size and yielded mixed results.^8,9,10^ As for other perinatal complications, one study found higher risk of preterm birth among individuals who reported food insecurity in pregnancy,^11^ and another reported no association between food insecurity in pregnancy and low birth weight.^12^

Food assistance programs such as the Supplemental Nutrition Assistance Program (SNAP) and Special Supplemental Nutrition Program for Individuals, Infants, and Children (WIC) aim to improve nutrition and health outcomes.^13,14,15^ However, studies among nonpregnant populations have shown mixed results on the role of these programs in the association between food insecurity and health outcomes,^16,17,18^ potentially due to residual confounding by socioeconomic status-related factors, as individuals enrolled in these programs are more likely to be vulnerable to poverty, food insecurity, and poor health.^19,20,21^ Nonetheless, such data in pregnancy are lacking.

To fill these knowledge gaps, our aims were 2-fold. First, we examined whether food insecurity in pregnancy was associated with the risk of maternal and neonatal complications. Second, we examined whether these potential associations differed by receipt of food assistance in pregnancy. We hypothesized that food insecurity in pregnancy was associated with increased risk of perinatal complications and that these associations were attenuated among individuals who received food assistance in pregnancy.

## Methods

### Study Design and Population

This is a cohort study using data from an online survey administered between June 22, 2020, and September 9, 2022 among members of Kaiser Permanente Northern California (KPNC) to evaluate health, health-related behaviors, and health care utilization in pregnancy during the COVID-19 pandemic.^22^ KPNC is an integrated health care system serving 4.6 million members in 21 hospitals and more than 255 outpatient clinics in 14 counties in Northern California. KPNC members are highly representative of the general population residing in the served geographic area in terms of age, sex, race, ethnicity, and neighborhood-level income and education.^23^

Detailed information on the study design is published elsewhere.^22^ Briefly, pregnant individuals at 12 or more gestational weeks who were aged 18 to 54 years and spoke English were identified through biweekly searches in the electronic health records (EHR) and recruited via email. At the study launch, postpartum individuals who delivered between January 1 and June 22, 2020, were also recruited to capture their experiences in pregnancy since the beginning of the COVID-19 pandemic. Of the 134 628 eligible individuals, 29 303 (21.8%) responded to the survey. For the current analysis, we excluded 1232 individuals (4.2%) with discontinued KPNC membership before delivery, 495 (1.7%) with multiple gestation, 284 (1.0%) with miscarriage, and 7954 (27.1%) with missing information on food insecurity in pregnancy, rendering an analytic sample of 19 338 individuals.

The KPNC institutional review board approved the study. Participants indicated informed consent by completing the survey after reviewing the consent information. The study followed the Strengthening the Reporting of Observational Studies in Epidemiology (STROBE) reporting guideline.

### Food Insecurity and Receipt of Food Assistance in Pregnancy

Food insecurity status since the beginning of the index pregnancy was assessed using the validated 2-item Hunger Vital Sign screener.^24^ Food insecurity in pregnancy (yes or no) was defined as responding often or sometimes true (vs never true) to at least 1 of 2 statements: (1) during their pregnancy they worried that their food would run out before they got money to buy, and (2) during their pregnancy the food they bought did not last and they did not have money to get more. The survey also asked whether the household received any food assistance (yes or no) since the beginning of the index pregnancy from any of the following: CalFresh or SNAP; California Food Access Program (CFAP) for qualified noncitizens; WIC; or other.

### Outcome Ascertainment

Information on the perinatal complications, including maternal (gestational diabetes, gestational hypertension, preeclampsia, and cesarean delivery) and neonatal (preterm birth, neonatal intensive care unit [NICU] admission, small-for-gestational age [SGA], and large-for-gestational age [LGA]), were obtained from the EHR as detailed elsewhere.^25^ Briefly, gestational diabetes with universal screening was diagnosed using the 2-step method according to the Carpenter and Coustan criteria as recommended by the American College of Obstetricians and Gynecologists (ACOG).^26^ Gestational hypertension and preeclampsia were diagnosed after 20 gestational weeks using physician diagnosis, antihypertensive medications, and/or blood pressure measurements according to the ACOG’s recommendations.^27^ Sex- and gestational age-specific birth weight categories were derived based on a 2017 US reference population as SGA (less than the 10th percentile) and LGA (more than the 90th percentile).^28^ Data about cesarean delivery, preterm birth (less than 37 weeks of gestation), and NICU admission were retrieved from the EHR. Adverse perinatal outcomes (APO), a composite outcome of maternal and neonatal complications, was defined as at least 1 of the following complications as done previously^29^: gestational diabetes, gestational hypertension, preeclampsia, preterm birth, NICU admission, and SGA infant.

### Covariates

The selection of covariates was informed by previous literature^30^ and by examining associations of covariates with food insecurity and perinatal complications. Selected covariates included age at delivery, race and ethnicity, neighborhood deprivation index (NDI), individual-level education, Medicaid or Medicare insurance during pregnancy, parity, and prepregnancy body mass index (BMI; calculated as weight in kilograms divided by height in meters squared).

Race and ethnicity, recognized as social constructs, were self-identified and categorized as Hispanic, non-Hispanic Asian or Pacific Islander, non-Hispanic Black, non-Hispanic White, and other (including American Indian or Alaska Native and multiracial, which were combined because of the small sample size). We geocoded the residential address of pregnant individuals obtained from the EHR at the time of survey completion and linked it to NDI, an indicator of neighborhood-level socioeconomic position that integrates census variables on education, occupation, housing, and income or poverty.^31^ We categorized NDI to quartiles based on its distribution in the entire KPNC membership during the study period. Prepregnancy BMI was calculated as prepregnancy weight in kilograms (measured within 12 weeks before pregnancy) divided by height in meters squared (measured within 12 months before pregnancy).

### Statistical Analyses

We used χ^2^ tests to compare participant characteristics by food insecurity status and receipt of food assistance in pregnancy. To examine associations of food insecurity in pregnancy with perinatal complications, we estimated adjusted relative risk (aRR) and 95% CI using modified Poisson regression models adjusted for covariates. Based on the literature,^16,17,18^ we tested our a priori hypothesis that the associations may differ by receipt of food assistance in pregnancy through calculating the *P* value for interaction using the Wald test and stratifying the analysis by receipt of food assistance.

We conducted sensitivity analyses to test the robustness of our findings. To account for survey nonresponse, we repeated our analysis using inverse probability weights calculated as described in a previous study.^32^ Acknowledging that NDI is a composite indicator that includes neighborhood-level education and that additionally adjusting for individual-level education may be an overadjustment, we ran a sensitivity analysis simultaneously adjusting for these 2 variables, NDI and individual-level education. Furthermore, we excluded individuals who completed the survey post partum. We also excluded individuals who reported that their household received food assistance in pregnancy only from other sources, which may include food banks or pantries with potentially different characteristics from the food assistance programs.

To account for missing data (ranging between less than 0.1% for NDI and 9.7% for education (Table), we used multiple imputation based on all covariates, exposure, and outcomes of interest to create 10 complete datasets and combined the analyses results on each complete dataset using the Rubin rule.^33^ *P* values were corrected for multiple comparison using the Benjamini-Hochberg procedure controlling the false discovery rate. All analyses were performed with SAS version 9.4 (SAS Institute). Statistical significance was set at *P *<* *.05. Data were analyzed from December 2023 to June 2024. All tests were 2-sided.

**Table.** Characteristics of 19 338 Individuals Who Responded to an Online Survey at Kaiser Permanente Northern California in 2020 to 2022 Overall and by Food Insecurity Status in Pregnancy

Characteristics	Participants, No. (%)		
	Food insecurity in pregnancy		Overall
	No	Yes	
No. (%)	16 631 (86.0)	2707 (14.0)	19 338 (100.0)
Age at delivery, y			
18-24	509 (3.1)	330 (12.2)	839 (4.3)
25-29	2597 (15.6)	716 (26.4)	3313 (17.1)
30-34	7098 (42.7)	922 (34.1)	8020 (41.5)
35-54	6427 (38.6)	739 (27.3)	7166 (37.1)
Race and ethnicity			
Asian or Pacific Islander	3734 (22.5)	690 (25.5)	4424 (22.9)
Black	478 (2.9)	202 (7.5)	680 (3.5)
Hispanic	2909 (17.5)	938 (34.7)	3847 (19.9)
White	8627 (51.9)	756 (27.9)	9383 (48.5)
Other^a^	553 (3.3)	79 (2.9)	632 (3.3)
Missing	330 (2.0)	42 (1.6)	372 (1.9)
Neighborhood deprivation index, quartile^b^			
1 (Least deprived)	5914 (35.6)	474 (17.5)	6388 (33.0)
2	4611 (27.7)	649 (24.0)	5260 (27.2)
3	3626 (21.8)	761 (28.1)	4387 (22.7)
4 (Most deprived)	2472 (14.9)	822 (30.4)	3294 (17.0)
Missing	8 (0)	1 (0)	9 (0)
Education			
High school or lower	975 (5.9)	488 (18.0)	1463 (7.6)
College, some or degree	8397 (50.5)	1649 (60.9)	10 046 (51.9)
Graduate degree	5639 (33.9)	319 (11.8)	5958 (30.8)
Missing	1620 (9.7)	251 (9.3)	1871 (9.7)
Medicaid or Medicare in pregnancy			
No	15 810 (95.1)	2203 (81.4)	18 013 (93.1)
Yes	801 (4.8)	492 (18.2)	1293 (6.7)
Missing	20 (0.1)	12 (0.4)	32 (0.2)
Prepregnancy BMI^c^			
Healthy weight	7466 (44.9)	795 (29.4)	8261 (42.7)
Overweight	4906 (29.5)	764 (28.2)	5670 (29.3)
Obesity	4108 (24.7)	1105 (40.8)	5213 (27.0)
Missing	151 (0.9)	43 (1.6)	194 (1.0)
Parity			
Nulliparous	7551 (45.4)	1049 (38.8)	8600 (44.5)
Multiparous	8356 (50.2)	1535 (56.7)	9891 (51.1)
Missing	724 (4.4)	123 (4.5)	847 (4.4)
Food assistance in pregnancy			
No	15 844 (95.3)	2023 (74.7)	17 867 (92.4)
Yes	787 (4.7)	684 (25.3)	1471 (7.6)

Abbreviation: BMI, body mass index.

^a^ Other race and ethnicity includes American Indian or Alaskan Native and multiracial individuals.

^b^ Neighborhood deprivation index is a validated composite score of US Census indicators of education, income and poverty, employment, housing, and occupation.

^c^ Racial- and ethnic-specific prepregnancy BMI categories derived as follows: for Black, Hispanic, and White individuals as well as those in the other race and ethnicity group: healthy weight (BMI<25.0), overweight (25.0-29.9), obesity (≥30.0); for Asian or Pacific Islander individuals: healthy weight (<23.0), overweight (23.0 to 27.4), and obesity (≥27.5).

## Results

### Characteristics of Study Population

Among 134 628 eligible individuals, 19 338 were included in the analysis (aged 30 to 34 years at delivery, 8020 individuals [41.5%]; 3847 Hispanic individuals [19.9%]; 9383 White individuals [48.5%]). Compared with individuals who were not included in the analysis, those who were included were more likely to be older at delivery, White, reside in the least deprived neighborhoods, have higher educational attainment, not have Medicaid or Medicare in pregnancy, be nulliparous, and have prepregnancy healthy weight (eTable 1 in Supplement 1).

Food insecurity in pregnancy was reported by 2707 individuals (14.0%) (Table). Individuals with vs without food insecurity in pregnancy were more likely to be younger at delivery, be Black or Hispanic, reside in the most deprived neighborhoods, have lower educational attainment of high school or lower, have Medicaid or Medicare in pregnancy, have prepregnancy obesity, be multiparous, and receive food assistance in pregnancy (Table).

**Figure 1.**
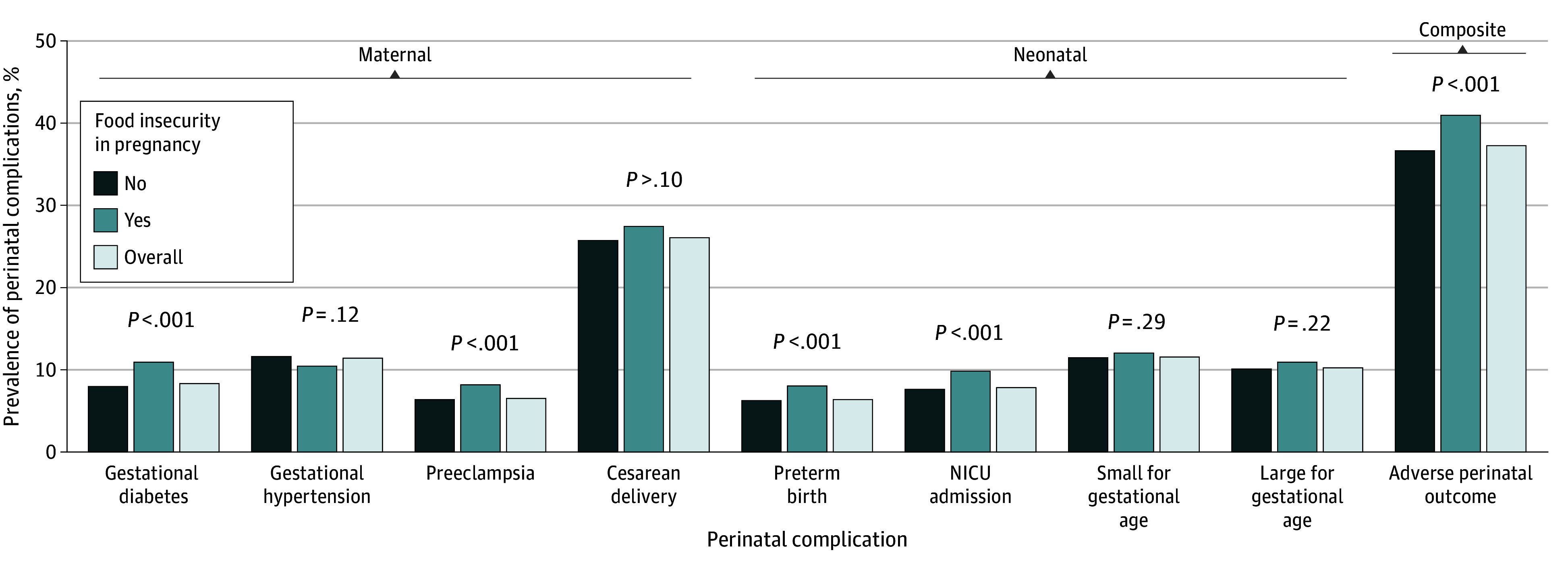
Prevalence of Perinatal Complications by Food Insecurity Status in Pregnancy Among 19 338 Individuals Who Responded to an Online Survey at Kaiser Permanente Northern California in 2020 to 2022 Adverse perinatal outcome is a composite outcome of gestational diabetes, gestational hypertension, preeclampsia, preterm birth, NICU (neonatal intensive care) admission, and small-for-gestational age infant. *P *values were FDR corrected. The *P_FDR_* values were calculated according to the Benjamini-Hochberg procedure controlling the false discovery rate and are presented above the relevant columns comparing no food insecurity vs food insecurity.

Individuals with vs without food insecurity in pregnancy had higher prevalence of gestational diabetes (295 [10.9%] vs 1314 [7.9%]), preeclampsia (219 [8.1%] vs 1048 [6.3%]), preterm birth (217 [8.0%] vs 1014 [6.1%]), NICU admission (265 [9.8%] vs 1247 [7.5%]), and APO (1113 [41.1%] vs 6120 [36.8%]) (*P* < .001) (Figure 1). The prevalence of gestational hypertension (287 [10.6%] vs 1946 [11.7%]; *P* = .12 ), cesarean delivery (744 [27.5%] vs 4291 [25.8%]; *P* = .10), SGA (328 [12.1%] vs 1913 [11.5%]; *P* = .29), and LGA (292 [10.8%] vs 1663 [10.0%]; *P* = .22) did not differ between individuals with vs without food insecurity in pregnancy.

A total of 1471 individuals (7.6%) reported that their household received food assistance in pregnancy, with the majority receiving assistance from WIC (824 [4.3%]), followed by CalFresh or SNAP (628 [3.3%]), and CFAP (19 [0.1%]). Furthermore, 302 individuals (1.2%) reported receiving assistance only from other sources. Individuals in households that received food assistance in pregnancy, compared with those in households that did not, were more likely to be younger at delivery, be Black or Hispanic, reside in the most deprived neighborhoods, have lower educational attainment, have Medicaid or Medicare in pregnancy, have prepregnancy obesity, be multiparous, and report food insecurity in pregnancy (684 [46.5%] vs 2023 [11.3%]) (eTable 2 in Supplement 1).

### Association of Food Insecurity in Pregnancy With Perinatal Complications

**Figure 2.**
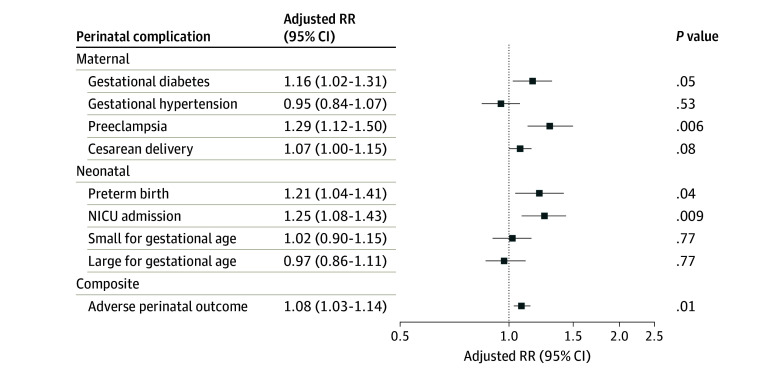
Association of Food Insecurity in Pregnancy With Perinatal Complications Among 19 338 Individuals Who Responded to an Online Survey at Kaiser Permanente Northern California in 2020 to 2022 Individuals without food insecurity served as the reference category. Models adjusted for age at delivery, race, ethnicity, neighborhood deprivation index, education, Medicaid or Medicare insurance during pregnancy, prepregnancy body mass index, parity, and receipt of food assistance in pregnancy. Adverse perinatal outcome is a composite outcome of gestational diabetes, gestational hypertension, preeclampsia, preterm birth, NICU admission, and small-for-gestational age infant. *P *values were FDR corrected. The *P_FDR_* values were calculated according to the Benjamini-Hochberg procedure controlling the false discovery rate. FDR indicates false discovery rate; NICU, neonatal intensive care unit; RR, relative risk.

After adjusting for covariates, individuals with vs without food insecurity in pregnancy had a higher risk of maternal and neonatal complications, specifically gestational diabetes (adjusted RR, 1.16 [95% CI, 1.02-1.31]), preeclampsia (aRR, 1.29 [95% CI, 1.12-1.50]), preterm birth (aRR, 1.21 [95% CI, 1.04-1.41]), and NICU admission (aRR, 1.25 [95% CI, 1.08-1.43]), and composite APO (aRR, 1.08 [95% CI, 1.03-1.14]) (Figure 2). There was no association of food insecurity in pregnancy with gestational hypertension (aRR, 0.95 [95% CI, 0.84-1.07]), cesarean delivery (aRR, 1.07 [95% CI, 1.00-1.15]), SGA (aRR, 1.02 [95% CI, 0.90-1.15]), and LGA (aRR, 0.97 [95% CI, 0.86-1.11]).

**Figure 3.**
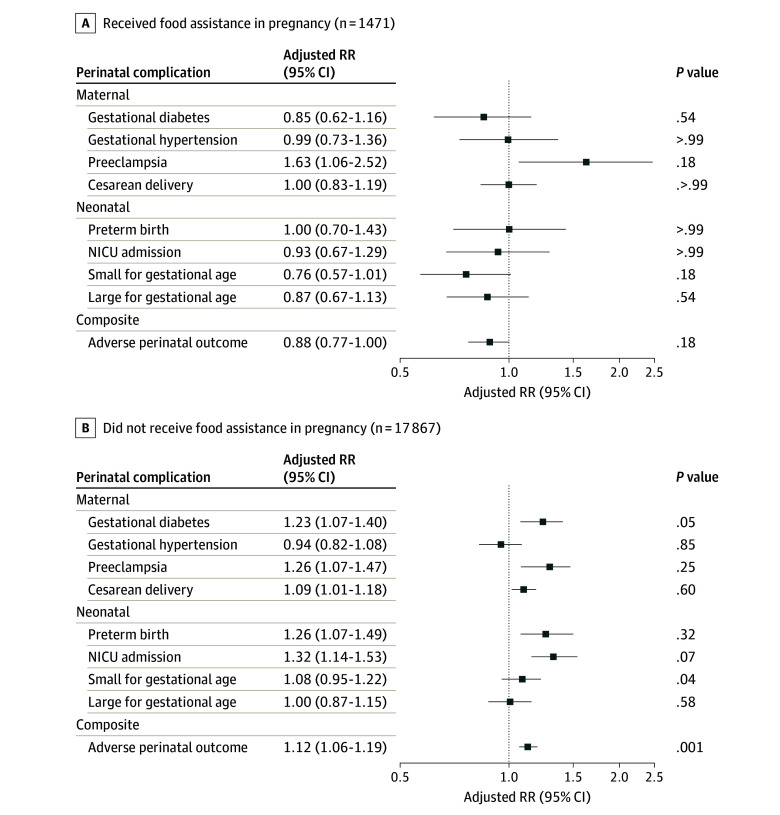
Association of Food Insecurity in Pregnancy With Perinatal Complications by Receipt of Food Assistance in Pregnancy Among 19 338 Individuals Who Responded to an Online Survey at Kaiser Permanente Northern California in 2020 to 2022 Adverse perinatal outcome is a composite outcome of gestational diabetes, gestational hypertension, preeclampsia, preterm birth, NICU admission, and small-for-gestational age infant. Individuals without food insecurity served as the reference category. Models adjusted for age at delivery, race, ethnicity, neighborhood deprivation index, education, Medicaid or Medicare insurance during pregnancy, prepregnancy body mass index, and parity. *P *values were FDR corrected. The *P* value for interaction between food insecurity in pregnancy and receipt of food assistance was calculated using the Wald test. The *P_FDR_* values were calculated according to the Benjamini-Hochberg procedure controlling the false discovery rate. FDR indicates false discovery rate; NICU, neonatal intensive care unit; RR, relative risk.

The associations differed by receipt of food assistance in pregnancy (Figure 3). Among individuals in households that received food assistance in pregnancy, associations of food insecurity in pregnancy with the risk of all perinatal complications were attenuated to the null, except for preeclampsia (aRR, 1.63 [95% CI, 1.06-2.52]). On the contrary, among individuals in households that did not receive food assistance in pregnancy, individuals with vs without food insecurity in pregnancy had a higher risk of gestational diabetes (aRR, 1.23 [95% CI, 1.07-1.40]), preeclampsia (aRR, 1.26 [95% CI, 1.07-1.47]), cesarean delivery (aRR, 1.09 [95% CI, 1.01-1.18]), preterm birth (aRR, 1.26 [95% CI, 1.07-1.49]), NICU admission (aRR, 1.32 [95% CI, 1.14-1.53]), and APO (1.12 [95% CI, 1.06-1.19]), but not gestational hypertension (aRR, 0.94 [95% CI, 0.82-1.08]), SGA (aRR, 1.08 [95% CI, 0.95-1.22]), or LGA (aRR, 1.00 [95% CI, 0.87-1.15]).

Sensitivity analysis using inverse probability weights to account for survey nonresponse yielded similar results (eTable 3 in Supplement 1). Furthermore, additionally adjusting for individual-level education yielded similar, although slightly attenuated, results (eTable 4 in Supplement 1). Another analysis limited to individuals (15 070 [77.9%]) who responded to the survey during pregnancy (mean [SD], 19.3 [7.2] gestation weeks), excluding those (4268 [22.1%]) who responded to the survey post partum (9.6 [6.6] postpartum weeks), yielded similar results (eTable 5 in Supplement 1). Furthermore, excluding individuals who reported that their household received food assistance in pregnancy only from other sources (226 [1.2%]) yielded similar results (eTable 6 in Supplement 1).

## Discussion

In a large, diverse population of individuals in Northern California, the prevalence of food insecurity in pregnancy was 14.0% in 2020 to 2022. We found that food insecurity in pregnancy was associated with a higher risk of maternal-related (gestational diabetes and preeclampsia) and neonatal-related (preterm birth and NICU admission) complications and a composite APO. The associations of food insecurity with the perinatal complications were overall attenuated to the null among individuals in households that received food assistance in pregnancy but persisted among those in households that did not receive assistance. Our findings provide evidence on the higher risk of perinatal complications among individuals with food insecurity in pregnancy and the effect modification of this association by receipt of food assistance in pregnancy.

The prevalence of food insecurity among pregnant individuals in the US remains unknown. The annual, nationally representative food insecurity surveillance conducted by the US Department of Agriculture (USDA) does not distinguish by pregnancy status. The Pregnancy Risk Assessment Monitoring System (PRAMS) estimated a 6.7% prevalence of food insecurity among pregnant individuals in 2020 across 14 states,^34^ and the National Health Interview Survey (NHIS) estimated a prevalence at 10.8% among pregnant and postpartum individuals in 2019 to 2021.^6^ Both estimates are lower than the 14.0% prevalence in our study in 2020 to 2022, which can be partially attributed to differences in timeframe, participant characteristics, and food insecurity scales used. The PRAMS study assessed food insecurity using a single question that likely reflects more severe forms of food insecurity, thus underestimating the prevalence of food insecurity.^34^ The NHIS study used the 10-item USDA Family Food Security questionnaire over a 30-day period covering pregnancy and/or post partum,^6^ whereas we used the validated 2-item Hunger Vital Sign screener and assessed food insecurity since the beginning of pregnancy.^24,35^

There is a paucity of research investigating the associations of food insecurity in pregnancy with perinatal complications. The association of food insecurity in pregnancy with gestational diabetes is unclear with mixed results, and the majority of studies were conducted with small sample sizes.^8,9,10^ While a study in Connecticut of 70 individuals revealed higher odds of gestational diabetes among pregnant individuals with food insecurity,^8^ the National Children’s Study in 7 states, which included 592 individuals, and the Pregnancy, Infection, and Nutrition cohort study in North Carolina, which included 810 individuals, reported null associations.^9,10^ There is a potential for misclassification of food insecurity status in the latter 2 studies as they assessed food insecurity over the previous 12 months,^9,10^ which may not reflect food insecurity in pregnancy. As for neonatal complications, a study of 268 individuals in Los Angeles showed that those who reported food insecurity in pregnancy had 3 times higher risk of preterm birth.^11^ On the other hand, the association of food insecurity in pregnancy with low birth weight was not significant using the PRAMS data of 50 915 live births from 11 states from 2009 to 2017.^12^ Less is known about the associations of food insecurity with other perinatal complications, including gestational hypertension, preeclampsia, cesarean delivery, and NICU admission.

The underlying mechanisms linking food insecurity in pregnancy with perinatal complications are complex and remain to be elucidated.^36^ One potential mechanism includes constrained food options, which may result in less healthy diets^36,37,38^ and significantly varying eating patterns due to inconsistent budgets.^39^ This may contribute to visceral adiposity, inadequate weight gain,^40^ and insulin resistance,^41^ which are associated with higher risk of perinatal complications like gestational diabetes.^10^ Nutrition insecurity has been recently proposed as a related concept, defined as a lack of consistent access to affordable, nutritious foods and beverages that support well-being and help prevent or manage disease.^42^ This construct emphasizes food quality, and it is intended to complement, rather than replace, traditional measures of food insecurity. Future research should investigate the association between nutrition insecurity and perinatal complications, emphasizing the crucial role of a healthy, high-quality diet during pregnancy.^43^ Another plausible mechanism is through the adverse impact of food insecurity on the pregnant individual’s mental health status, including depression and anxiety, that may contribute to dysregulation of the hypothalamic-pituitary-adrenal axis, metabolic disturbance, and inflammation, which in turn increase the risk of perinatal complications.^44,45^

We found that the association of food insecurity in pregnancy with perinatal complications was overall attenuated to the null among individuals who received food assistance in pregnancy. WIC was the most reported food assistance program among our study population. Despite intensive research, the association between WIC and pregnancy outcomes remain controversial,^21^ which led to different policy proposals on WIC funds, including the recent budget shortfalls in 2024.^46,47,48,49^ The self-selection bias into food assistance programs makes it difficult to assess study associations between these programs and health outcomes.^19,20^ However, after adjusting for neighborhood- and individual-level socioeconomic characteristics, we found that individuals with food insecurity who did not receive food assistance in pregnancy had the highest risk of perinatal complications. Considering that food assistance programs, such as WIC, can enhance health outcomes for mother-child dyads by providing nutritious food packages, nutrition counseling, and necessary referrals to health care professionals,^50^ our research supports policies designed to ensure these programs are adequately funded and accessible to a broader population. Additionally, in alignment with ACOG’s recommendations,^51^ our findings endorse screening for food insecurity during pregnancy and referral to food assistance programs as part of prenatal care, recognizing that pregnancy is a crucial period for health across the lifespan. These insights may address the current gap in evidence identified by the US Preventive Services Task Force regarding the screening for food insecurity in primary care settings.^52^

### Strengths and Limitations

Our study has several strengths. We assessed food insecurity since the beginning of the index pregnancy using the Hunger Vital Sign screener, a 2-item validated food insecurity screening tool with 97% sensitivity.^24,35^ Furthermore, we extracted data on perinatal complications from the EHR, as opposed to self-report in most previous studies.

This study has limitations. While the underlying population from which this study sample was selected is sociodemographically diverse,^23^ generalizability to other populations may be limited since our study sample resided in Northern California. Furthermore, individuals included in the analysis were more likely to be younger, White individuals, reside in less deprived neighborhoods, not have Medicaid or Medicare insurance during pregnancy, and have healthy weight compared with individuals who were not included in the analysis; however, in a sensitivity analysis using inverse probability weighting to account for these differences, we observed similar results to the main analysis. Although we accounted for socioeconomic status in our analysis by adjusting for NDI, a multidimensional index that includes neighborhood-level factors such as wealth and income, education, occupation, and housing conditions, we cannot rule out the possibility of residual confounding by unmeasured covariates such as individual-level income. Finally, 4271 individuals (22.1%) responded to the survey post partum, which may introduce recall bias; however, in a sensitivity analysis limited to individuals who responded to the survey in pregnancy, we observed similar results.

## Conclusions

This study highlights associations of food insecurity in pregnancy with higher risk of perinatal complications, with attenuated associations among individuals who received food assistance programs in pregnancy. Additional research is needed to confirm our findings in other study populations and inform evidence-based interventions to improve health outcomes among pregnant individuals with food insecurity and their offspring. Future efforts should build on these findings to enhance screening and management of food insecurity in pregnancy, which is in line with ACOG’s recommendation that health care clinicians screen pregnant individuals for food insecurity among other social determinants of health.^51^ Public health efforts and policies should support food assistance programs and ensure wider participation to help address food insecurity in pregnancy as a step forward to ensure equitable prenatal care and pregnancy outcomes.

## Notes

Supplement 1. **eTable 1.** Characteristics of Individuals Included vs Not Included in the Study **eTable 2.** Characteristics of 19 338 Individuals Who Responded to an Online Survey at Kaiser Permanente Northern California in 2020 to 2022 Overall and by Receipt of Food Assistance **eTable 3.** Association of Food Insecurity in Pregnancy With Perinatal Complications Using Inverse Probability Weights to Account For Survey Nonresponse Among 19 338 Individuals Who Responded to an Online Survey in Pregnancy at Kaiser Permanente Northern California in 2020 to 2022 **eTable 4.** Association of Food Insecurity in Pregnancy With Perinatal Complications Additionally Adjusting for Individual-Level Education Among 19 338 Individuals Who Responded to an Online Survey in Pregnancy at Kaiser Permanente Northern California in 2020 to 2022 **eTable 5.** Association of Food Insecurity in Pregnancy With Perinatal Complications Among 15 070 Individuals Who Responded to an Online Survey in Pregnancy at Kaiser Permanente Northern California in 2020 to 2022 **eTable 6.** Association of Food Insecurity in Pregnancy With Perinatal Complications Among 19 112 Individuals Who Responded to an Online Survey at Kaiser Permanente Northern California in 2020 to 2022 Excluding Those Who Reported Receiving Food Assistance Only From Other Sources
eReferences

Supplement 2.
Data Sharing Statement

## References

[1] Economic Research Service US Department of Agriculture. Definitions of food security. Accessed January 31, 2024. https://www.ers.usda.gov/topics/food-nutrition-assistance/food-security-in-the-u-s/definitions-of-food-security/

[2] Palakshappa D, Garg A, Peltz A, Wong CA, Cholera R, Berkowitz SA. Food insecurity was associated with greater family health care expenditures in the US, 2016-2017. Health Aff (Millwood). 2023;42(1):44-52. doi:10.1377/hlthaff.2022.00414 36623217 PMC10926282

[3] Ma H, Wang X, Li X, . Food insecurity and premature mortality and life expectancy in the US. JAMA Intern Med. 2024;184(3):301-310. doi:10.1001/jamainternmed.2023.7968 38285593 PMC10825785

[4] Rabbitt MP, Hales LJ, Burke MP, Coleman-Jensen A. Household food security in the United States in 2022. Economic Research Service. Accessed December 11, 2024. https://www.ers.usda.gov/publications/pub-details/?pubid=107702

[5] Dolin CD, Compher CC, Oh JK, Durnwald CP. Pregnant and hungry: addressing food insecurity in pregnant women during the COVID-19 pandemic in the United States. Am J Obstet Gynecol MFM. 2021;3(4):100378. doi:10.1016/j.ajogmf.2021.100378 33932628 PMC9751596

[6] Ujah OI, Olaore P, Ogbu CE, Kirby RS. Trends, prevalence, and risk factors of food insecurity among pregnant and postpartum women in the United States: findings from the 2019 to 2021 National Health Interview Survey. J Womens Health (Larchmt). 2023;32(10):1096-1103. doi:10.1089/jwh.2023.0250 37579074

[7] Seligman HK, Laraia BA, Kushel MB. Food insecurity is associated with chronic disease among low-income NHANES participants. J Nutr. 2010;140(2):304-310. doi:10.3945/jn.109.112573 20032485 PMC2806885

[8] Cooper S, Graham M, Kuo CL, Khangura R, Schmidt A, Bakaysa S. The relationship between food security and gestational diabetes among pregnant women. AJP Rep. 2022;12(3):e131-e138. doi:10.1055/s-0042-1751082 36034747 PMC9410985

[9] Richards M, Weigel M, Li M, Rosenberg M, Ludema C. Food insecurity, gestational weight gain and gestational diabetes in the National Children’s Study, 2009-2014. J Public Health (Oxf). 2021;43(3):558-566. doi:10.1093/pubmed/fdaa093 32618341

[10] Laraia BA, Siega-Riz AM, Gundersen C. Household food insecurity is associated with self-reported pregravid weight status, gestational weight gain, and pregnancy complications. J Am Diet Assoc. 2010;110(5):692-701. doi:10.1016/j.jada.2010.02.014 20430130 PMC3018748

[11] Sandoval VS, Jackson A, Saleeby E, Smith L, Schickedanz A. Associations between prenatal food insecurity and prematurity, pediatric health care utilization, and postnatal social needs. Acad Pediatr. 2021;21(3):455-461. doi:10.1016/j.acap.2020.11.020 33253934 PMC8026536

[12] De Silva DA, Thoma ME, Anderson EA, Kim J. Infant sex-specific associations between prenatal food insecurity and low birth weight: a multistate analysis. J Nutr. 2022;152(6):1538-1548. doi:10.1093/jn/nxac062 35265994

[13] Metallinos-Katsaras E, Gorman KS, Wilde P, Kallio J. A longitudinal study of WIC participation on household food insecurity. Matern Child Health J. 2011;15(5):627-633. doi:10.1007/s10995-010-0616-5 20455015

[14] Soneji S, Beltrán-Sánchez H. Association of special supplemental nutrition program for women, infants, and children with preterm birth and infant mortality. JAMA Netw Open. 2019;2(12):e1916722. doi:10.1001/jamanetworkopen.2019.16722 31800070 PMC6902759

[15] Committee on Examination of the Adequacy of Food Resources and SNAP Allotments; Food and Nutrition Board; Committee on National Statistics; Institute of Medicine; National Research Council. Supplemental nutrition assistance program: examining the evidence to define benefit adequacy. Caswell JA, Yaktine AL, eds. National Academies Press; 2013. 24901188

[16] Pryor L, Melchior M, Avendano M, Surkan PJ. Childhood food insecurity, mental distress in young adulthood and the supplemental nutrition assistance program. Prev Med. 2023;168:107409. doi:10.1016/j.ypmed.2022.107409 36592677

[17] Leung CW, Epel ES, Willett WC, Rimm EB, Laraia BA. Household food insecurity is positively associated with depression among low-income supplemental nutrition assistance program participants and income-eligible nonparticipants. J Nutr. 2015;145(3):622-627. doi:10.3945/jn.114.199414 25733480

[18] Leung CW, Fulay AP, Parnarouskis L, Martinez-Steele E, Gearhardt AN, Wolfson JA. Food insecurity and ultra-processed food consumption: the modifying role of participation in the Supplemental Nutrition Assistance Program (SNAP). Am J Clin Nutr. 2022;116(1):197-205. doi:10.1093/ajcn/nqac049 35199832 PMC9257471

[19] Nord M. Does SNAP decrease food insecurity?: Untangling the self-selection effect. Vol 85. DIANE Publishing; 2009.

[20] Bitler M. The health and nutrition effects of SNAP: selection into the program and a review of the literature on its effects. Accessed June 27, 2024. https://www.ukcpr.org/Publications/DP2014-02.pdf

[21] Yan J. Is WIC effective in improving pregnancy-related outcomes? an empirical reassessment. Econ Hum Biol. 2022;47:101197. doi:10.1016/j.ehb.2022.101197 36427408

[22] Ames JL, Ferrara A, Avalos LA, . COVID-19 prevalence, symptoms, and sociodemographic disparities in infection among insured pregnant women in Northern California. PLoS One. 2021;16(9):e0256891. doi:10.1371/journal.pone.0256891 34478463 PMC8415576

[23] Davis AC, Voelkel JL, Remmers CL, Adams JL, McGlynn EA. Comparing Kaiser Permanente members to the general population: implications for generalizability of research. Perm J. 2023;27(2):87-98. doi:10.7812/TPP/22.172 37170584 PMC10266863

[24] Gundersen C, Engelhard EE, Crumbaugh AS, Seligman HK. Brief assessment of food insecurity accurately identifies high-risk US adults. Public Health Nutr. 2017;20(8):1367-1371. doi:10.1017/S1368980017000180 28215190 PMC10261547

[25] Chehab RF, Ferrara A, Greenberg MB, Ngo AL, Feng J, Zhu Y. Glycemic control trajectories and risk of perinatal complications among individuals with gestational diabetes. JAMA Netw Open. 2022;5(9):e2233955. doi:10.1001/jamanetworkopen.2022.33955 36173631 PMC9523493

[26] Carpenter MW, Coustan DR. Criteria for screening tests for gestational diabetes. Am J Obstet Gynecol. 1982;144(7):768-773. doi:10.1016/0002-9378(82)90349-0 7148898

[27] American College of Obstetricians and Gynecologists. Gestational hypertension and preeclampsia: ACOG practice bulletin, number 222. Obstet Gynecol. 2020;135(6):e237-e260. doi:10.1097/AOG.0000000000003891 32443079

[28] Aris IM, Kleinman KP, Belfort MB, Kaimal A, Oken E. A 2017 US reference for singleton birth weight percentiles using obstetric estimates of gestation. Pediatrics. 2019;144(1). doi:10.1542/peds.2019-0076 PMC6615520 31201230

[29] Makarem N, Chau K, Miller EC, . Association of a Mediterranean diet pattern with adverse pregnancy outcomes among US women. JAMA Netw Open. 2022;5(12):e2248165. doi:10.1001/jamanetworkopen.2022.48165 36547978 PMC9857221

[30] Bastian A, Parks C, Yaroch A, . Factors associated with food insecurity among pregnant women and caregivers of children aged 0-6 years: a scoping review. Nutrients. 2022;14(12):2407. doi:10.3390/nu14122407 35745136 PMC9227310

[31] Codebook Neighborhood Deprivation Index Data. National Cancer Institute GIS Portal for Cancer Research. Accessed August 2023. https://www.gis.cancer.gov/research/NeighDeprvIndex_Codebook.pdf

[32] Avalos LA, Nance N, Badon SE, . Associations of COVID-19-related health, healthcare and economic factors with prenatal depression and anxiety. Int J Public Health. 2022;67:1604433. doi:10.3389/ijph.2022.1604433 35601595 PMC9114304

[33] Rubin DB, Schenker N. Multiple imputation in health-care databases: an overview and some applications. Stat Med. 1991;10(4):585-598. doi:10.1002/sim.4780100410 2057657

[34] Hinkle SN, Dolin CD, Keddem S, Kinsey EW. Patterns in food insecurity during pregnancy, 2004 to 2020. JAMA Netw Open. 2023;6(7):e2324005. doi:10.1001/jamanetworkopen.2023.24005 37462976 PMC10354677

[35] Hager ER, Quigg AM, Black MM, . Development and validity of a 2-item screen to identify families at risk for food insecurity. Pediatrics. 2010;126(1):e26-e32. doi:10.1542/peds.2009-3146 20595453

[36] Weiser SD, Palar K, Hatcher AM, Young S, Frongillo EA, Laraia B. Food insecurity and health: a conceptual framework. Food insecurity and public health. CRC Press; 2015:23-50. doi:10.1201/b18451-3

[37] Tarasuk VS. Household food insecurity with hunger is associated with women’s food intakes, health and household circumstances. J Nutr. 2001;131(10):2670-2676. doi:10.1093/jn/131.10.2670 11584089

[38] Tarasuk VS, Beaton GH. Women’s dietary intakes in the context of household food insecurity. J Nutr. 1999;129(3):672-679. doi:10.1093/jn/129.3.672 10082773

[39] Tarasuk V, McIntyre L, Li J. Low-income women’s dietary intakes are sensitive to the depletion of household resources in one month. J Nutr. 2007;137(8):1980-1987. doi:10.1093/jn/137.8.1980 17634274

[40] Crandall AK, Temple JL, Kong KL. The association of food insecurity with the relative reinforcing value of food, BMI, and gestational weight gain among pregnant women. Appetite. 2020;151:104685. doi:10.1016/j.appet.2020.104685 32229225 PMC9540643

[41] Laraia BA. Food insecurity and chronic disease. Adv Nutr. 2013;4(2):203-212. doi:10.3945/an.112.003277 23493536 PMC3649100

[42] Mozaffarian D, Fleischhacker S, Andrés JR. Prioritizing nutrition security in the US. JAMA. 2021;325(16):1605-1606. doi:10.1001/jama.2021.1915 33792612

[43] Marshall NE, Abrams B, Barbour LA, . The importance of nutrition in pregnancy and lactation: lifelong consequences. Am J Obstet Gynecol. 2022;226(5):607-632. doi:10.1016/j.ajog.2021.12.035 34968458 PMC9182711

[44] Augusto ALP, de Abreu Rodrigues AV, Domingos TB, Salles-Costa R. Household food insecurity associated with gestacional and neonatal outcomes: a systematic review. BMC Pregnancy Childbirth. 2020;20(1):229. doi:10.1186/s12884-020-02917-9 32303221 PMC7164154

[45] Laraia B, Vinikoor-Imler LC, Siega-Riz AM. Food insecurity during pregnancy leads to stress, disordered eating, and greater postpartum weight among overweight women. Obesity (Silver Spring). 2015;23(6):1303-1311. doi:10.1002/oby.21075 25959858 PMC6563905

[46] Currie J, Rajani I. Within-mother estimates of the effects of wic on birth outcomes in New York City. Econ Inq. 2015;53(4):1691-1701. doi:10.1111/ecin.12219 28503006 PMC5425167

[47] Ludwig J, Miller M. Interpreting the WIC debate. J Policy Anal Manage. 2005;24(4):691-701. doi:10.1002/pam.20133 16201055

[48] Joyce T, Racine A, Yunzal-Butler C. Reassessing the WIC effect: evidence from the pregnancy nutrition surveillance system. J Policy Anal Manage. 2008;27(2):277-303. doi:10.1002/pam.20325 18401924

[49] Following topline budget agreement: Congress must act to fully fund WIC in 2024. US Department of Agriculture. Accessed February 14, 2024. https://www.usda.gov/media/press-releases/2024/01/11/following-topline-budget-agreement-congress-must-act-fully-fund-wic

[50] USDA. About WIC: how WIC helps. January 29, 2024. https://www.fns.usda.gov/wic/about-wic-how-wic-helps

[51] Committee on Health Care for Underserved Women. ACOG Committee Opinion No. 729: importance of social determinants of health and cultural awareness in the delivery of reproductive health care. Obstet Gynecol. 2018;131(1):e43-e48. doi:10.1097/AOG.0000000000002459 29266079

[52] U.S. Preventive Services Task Force. Draft Recommendation Statement Food Insecurity: Screening. US Preventive Services Task Force. Accessed July 16, 2024. https://www.uspreventiveservicestaskforce.org/uspstf/draft-recommendation/food-insecurity-preventive-services

