# Supplementary material for: Food Insecurity in Pregnancy, Receipt of Food Assistance, and Perinatal Complications
Source: JAMA Netw Open. 2025 Jan 23;8(1):e2455955. doi: 10.1001/jamanetworkopen.2024.55955 (PMC11758595; doi:10.1001/jamanetworkopen.2024.55955)
Supplement: Supplement 1. — eTable 1. Characteristics of Individuals Included vs Not Included in the Study eTable 2. Characteristics of 19 338 Individuals Who Responded to an Online Survey at Kaiser Permanente Northern California in 2020 to 2022 Overall and by Receipt of Food Assistance eTable 3. Association of Food Insecurity in Pregnancy With Perinatal Complications Using Inverse Probability Weights to Account For Survey Nonresponse Among 19 338 Individuals Who Responded to an Online Survey in Pregnancy at Kaiser Permanente Northern California in 2020 to 2022 eTable 4. Association of Food Insecurity in Pregnancy With Perinatal Complications Additionally Adjusting for Individual-Level Education Among 19 338 Individuals Who Responded to an Online Survey in Pregnancy at Kaiser Permanente Northern California in 2020 to 2022 eTable 5. Association of Food Insecurity in Pregnancy With Perinatal Complications Among 15 070 Individuals Who Responded to an Online Survey in Pregnancy at Kaiser Permanente Northern California in 2020 to 2022 eTable 6. Association of Food Insecurity in Pregnancy With Perinatal Complications Among 19 112 Individuals Who Responded to an Online Survey at Kaiser Permanente Northern California in 2020 to 2022 Excluding Those Who Reported Receiving Food Assistance Only From Other Sources eReferences [file jamanetwopen-e2455955-s001.pdf]

Supplemental Online Content

Chehab RF, Croen LA, Laraia BA, et al. Food insecurity in pregnancy, receipt of food assistance, and perinatal complications. *JAMA Netw Open*. 2025;8(1):e2455955. doi:10.1001/jamanetworkopen.2024.55955

eTable 1. Characteristics of Individuals Included vs Not Included in the Study

eTable 2. Characteristics of 19 338 Individuals Who Responded to an Online Survey at Kaiser Permanente Northern California in 2020 to 2022 Overall and by Receipt of Food Assistance

eTable 3. Association of Food Insecurity in Pregnancy With Perinatal Complications Using Inverse Probability Weights to Account For Survey Nonresponse Among 19 338 Individuals Who Responded to an Online Survey in Pregnancy at Kaiser Permanente Northern California in 2020 to 2022

eTable 4. Association of Food Insecurity in Pregnancy With Perinatal Complications Additionally Adjusting for Individual-Level Education Among 19 338 Individuals Who Responded to an Online Survey in Pregnancy at Kaiser Permanente Northern California in 2020 to 2022

eTable 5. Association of Food Insecurity in Pregnancy With Perinatal Complications Among 15 070 Individuals Who Responded to an Online Survey in Pregnancy at Kaiser Permanente Northern California in 2020 to 2022

eTable 6. Association of Food Insecurity in Pregnancy With Perinatal Complications Among 19 112 Individuals Who Responded to an Online Survey at Kaiser Permanente Northern California in 2020 to 2022 Excluding Those Who Reported Receiving Food Assistance Only From Other Sources

eReferences

This supplemental material has been provided by the authors to give readers additional information about their work.

© 2025 Chehab RF et al. *JAMA Network Open*.

eTable 1. Characteristics of Individuals Included vs Not Included in the Study

Characteristics	Included in the study		Overall
	No	Yes	
	n (%)		
	115290 (85.6)	19338 (14.4)	134628 (100.0)
**Age at delivery, years**			
18-24	12294 (10.7)	839 (4.3)	13133 (9.8)
25-29	28055 (24.3)	3313 (17.1)	31368 (23.3)
30-34	42312 (36.7)	8020 (41.5)	50332 (37.4)
35-54	32629 (28.3)	7166 (37.1)	39795 (29.6)
**Race and ethnicity**			
Asian/Pacific Islander	30715 (26.6)	4424 (22.9)	35139 (26.1)
Black	8234 (7.1)	680 (3.5)	8914 (6.6)
Hispanic	34910 (30.3)	3847 (19.9)	38757 (28.8)
White	35160 (30.5)	9383 (48.5)	44543 (33.1)
Other[a]	3240 (2.8)	632 (3.3)	3872 (2.9)
Missing	3031 (2.6)	372 (1.9)	3403 (2.5)
**Neighborhood deprivation index[b]**			
Quartile 1 (least deprived)	21255 (18.4)	6388 (33.0)	27643 (20.5)
Quartile 2	30542 (26.5)	5260 (27.2)	35802 (26.6)
Quartile 3	33551 (29.1)	4387 (22.7)	37938 (28.2)
Quartile 4 (most deprived)	29868 (25.9)	3294 (17.0)	33162 (24.6)
Missing	74 (0.1)	9 (0.0)	83 (0.1)
**Education**			
High school or lower	22254 (19.3)	1463 (7.6)	23717 (17.6)
College, some or degree	60453 (52.4)	10046 (51.9)	70499 (52.4)
Graduate degree	18685 (16.2)	5958 (30.8)	24643 (18.3)
Missing	13898 (12.1)	1871 (9.7)	15769 (11.7)
**Medicaid/Medicare in pregnancy**			
No	99307 (86.1)	18013 (93.1)	117320 (87.1)
Yes	14272 (12.4)	1293 (6.7)	15565 (11.6)
Missing	1711 (1.5)	32 (0.2)	1743 (1.3)
**Pre-pregnancy BMI[c]**			
Healthy weight	39798 (34.5)	8261 (42.7)	48059 (35.7)
Overweight	34350 (29.8)	5670 (29.3)	40020 (29.7)
Obesity	36797 (31.9)	5213 (27.0)	42010 (31.2)
Missing	4345 (3.8)	194 (1.0)	4539 (3.4)
**Parity**			
Nulliparous	46468 (40.3)	8600 (44.5)	55068 (40.9)
Multiparous	65409 (56.7)	9891 (51.1)	75300 (55.9)
Missing	3413 (3.0)	847 (4.4)	4260 (3.2)

© 2025 Chehab RF et al. *JAMA Network Open*.

BMI, body mass index.
[a]Other race and ethnicity includes American Indian/Alaskan Native and Multiracial individuals.
[b]Neighborhood deprivation index is a validated composite score of US Census indicators of education, income and poverty, employment, housing, and occupation.
[c]Racial- and ethnic-specific pre-pregnancy BMI categories derived as follows: for non-Asian/Pacific Islander individuals: healthy weight (BMI <25.0 kg/m^2), overweight (25.0–29.9 kg/m^2), obesity (≥30.0 kg/m^2); for Asian/Pacific Islander individuals: healthy weight (<23.0 kg/m^2), overweight (23.0–27.4 kg/m^2), and obesity (≥27.5 kg/m^2).

© 2025 Chehab RF et al. *JAMA Network Open*.

eTable 2. Characteristics of 19 338 Individuals Who Responded to an Online Survey at Kaiser Permanente Northern California in 2020 to 2022 Overall and by Receipt of Food Assistance

	Received food assistance in pregnancy			Did not receive food assistance in pregnancy		
	Food insecurity in pregnancy			Food insecurity in pregnancy		
	No	Yes	Overall	No	Yes	Overall
	n (%)					
**Characteristics**	787 (53.5)	684 (46.5)	1471 (100.0)	15844 (88.7)	2023 (11.3)	17867 (100.0)
**Age at delivery, years**						
18-24	94 (11.9)	120 (17.5)	214 (14.5)	415 (2.6)	210 (10.4)	625 (3.5)
25-29	223 (28.3)	191 (27.9)	414 (28.1)	2374 (15.0)	525 (26.0)	2899 (16.2)
30-34	257 (32.7)	205 (30.0)	462 (31.4)	6841 (43.2)	717 (35.4)	7558 (42.3)
35-54	213 (27.1)	168 (24.6)	381 (25.9)	6214 (39.2)	571 (28.2)	6785 (38.0)
**Race and ethnicity**						
Asian/Pacific Islander	125 (15.9)	105 (15.4)	230 (15.6)	3609 (22.8)	585 (28.9)	4194 (23.5)
Black	69 (8.8)	86 (12.6)	155 (10.5)	409 (2.6)	116 (5.7)	525 (2.9)
Hispanic	317 (40.3)	307 (44.9)	624 (42.4)	2592 (16.4)	631 (31.2)	3223 (18.0)
White	238 (30.2)	157 (23.0)	395 (26.9)	8389 (52.9)	599 (29.6)	8988 (50.3)
Other[a]	27 (3.4)	18 (2.6)	45 (3.1)	526 (3.3)	61 (3.0)	587 (3.3)
Missing	11 (1.4)	11 (1.6)	22 (1.5)	319 (2.0)	31 (1.5)	350 (2.0)
**Neighborhood deprivation index[b]**						
Quartile 1 (least deprived)	92 (11.7)	62 (9.1)	154 (10.5)	5822 (36.7)	412 (20.4)	6234 (34.9)
Quartile 2	164 (20.8)	118 (17.3)	282 (19.2)	4447 (28.1)	531 (26.2)	4978 (27.9)
Quartile 3	218 (27.7)	203 (29.7)	421 (28.6)	3408 (21.5)	558 (27.6)	3966 (22.2)
Quartile 4 (most deprived)	313 (39.8)	300 (43.9)	613 (41.7)	2159 (13.6)	522 (25.8)	2681 (15.0)
Missing	0 (0.0)	1 (0.1)	1 (0.1)	8 (0.1)	0 (0.0)	8 (0.0)
**Education**						
High school or lower	171 (21.7)	184 (26.9)	355 (24.1)	804 (5.1)	304 (15.0)	1108 (6.2)

© 2025 Chehab RF et al. *JAMA Network Open*.

College, some or degree	483 (61.4)	418 (61.1)	901 (61.3)	7914 (49.9)	1231 (60.9)	9145 (51.2)
Graduate degree	54 (6.9)	31 (4.5)	85 (5.8)	5585 (35.2)	288 (14.2)	5873 (32.9)
Missing	79 (10.0)	51 (7.5)	130 (8.8)	1541 (9.7)	200 (9.9)	1741 (9.7)
**Medicaid/ Medicare in pregnancy**						
No	501 (63.7)	379 (55.4)	880 (59.8)	15309 (96.6)	1824 (90.2)	17133 (95.9)
Yes	282 (35.8)	300 (43.9)	582 (39.6)	519 (3.3)	192 (9.5)	711 (4.0)
Missing	4 (0.5)	5 (0.7)	9 (0.6)	16 (0.1)	7 (0.3)	23 (0.1)
**Pre-pregnancy BMI[c]**						
Healthy weight	211 (26.8)	163 (23.8)	374 (25.4)	7255 (45.8)	632 (31.2)	7887 (44.1)
Overweight	249 (31.6)	185 (27.0)	434 (29.5)	4657 (29.4)	579 (28.6)	5236 (29.3)
Obesity	312 (39.6)	325 (47.5)	637 (43.3)	3796 (24.0)	780 (38.6)	4576 (25.6)
Missing	15 (1.9)	11 (1.6)	26 (1.8)	136 (0.9)	32 (1.6)	168 (0.9)
**Parity**						
Nulliparous	163 (20.7)	169 (24.7)	332 (22.6)	7388 (46.6)	880 (43.5)	8268 (46.3)
Multiparous	606 (77.0)	497 (72.7)	1103 (75.0)	7750 (48.9)	1038 (51.3)	8788 (49.2)
Missing	18 (2.3)	18 (2.6)	36 (2.4)	706 (4.5)	105 (5.2)	811 (4.5)

BMI, body mass index.
[a]Other race and ethnicity includes American Indian/Alaskan Native and Multiracial individuals.
[b]Neighborhood deprivation index is a validated composite score of US Census indicators of education, income and poverty, employment, housing, and occupation.
[c]Racial- and ethnic-specific pre-pregnancy BMI categories derived as follows: for non-Asian/Pacific Islander individuals: healthy weight (BMI <25.0 kg/m^2), overweight (25.0–29.9 kg/m^2), obesity (≥30.0 kg/m^2); for Asian/Pacific Islander individuals: healthy weight (<23.0 kg/m^2), overweight (23.0–27.4 kg/m^2), and obesity (≥27.5 kg/m^2).

© 2025 Chehab RF et al. *JAMA Network Open*.

eTable 3. Association of Food Insecurity in Pregnancy With Perinatal Complications Using Inverse Probability Weights[a] to Account For Survey Nonresponse Among 19 338 Individuals Who Responded to an Online Survey in Pregnancy at Kaiser Permanente Northern California in 2020 to 2022

**Complications**	**Overall[b] (n= 19338)**	**Received food assistance in pregnancy[c] (n= 1471)**	**Did not receive food assistance in pregnancy[c] (n= 17867)**
	**Adjusted RR (95% CI)**		
**Maternal**			
Gestational diabetes	1.14 (1.00-1.31)	0.93 (0.67-1.30)	1.20 (1.03-1.39)
Gestational hypertension	0.96 (0.84-1.11)	0.95 (0.67-1.35)	0.96 (0.82-1.12)
Pre-eclampsia	1.32 (1.12-1.56)	1.54 (0.96-2.47)	1.29 (1.08-1.54)
Cesarean delivery	1.07 (0.99-1.16)	1.04 (0.86-1.26)	1.08 (0.99-1.18)
**Neonatal**			
Preterm birth	1.18 (1.00-1.41)	0.91 (0.62-1.34)	1.28 (1.07-1.53)
NICU admission	1.15 (0.98-1.35)	0.90 (0.64-1.28)	1.23 (1.04-1.45)
Small-for-gestational age	1.01 (0.88-1.15)	0.76 (0.56-1.05)	1.09 (0.94-1.25)
Large-for-gestational age	0.99 (0.86-1.14)	0.93 (0.70-1.23)	1.00 (0.85-1.17)
**Composite**			
Adverse perinatal outcome	1.07 (1.01-1.13)	0.88 (0.76-1.02)	1.12 (1.05-1.19)

CI, confidence interval; NICU, neonatal intensive care unit; RR, relative risk.
Adverse perinatal outcome is a composite outcome of gestational diabetes, gestational hypertension, pre-eclampsia, preterm birth, NICU admission, and small-for-gestational age infant.
Individuals without food insecurity served as the reference category.
[a]Inverse probability weights were calculated as described in a previous study[1] as inverse of predicted probabilities from logistic regression models that included variables predicted to be associated with survey response and with perinatal complications (age at delivery, race, ethnicity, neighborhood deprivation index, education, Medicaid/Medicare insurance in pregnancy, pre-pregnancy body mass index, and parity).
[b]Models adjusted for age at delivery, race, ethnicity, neighborhood deprivation index, individual-level education, Medicaid/Medicare insurance in pregnancy, pre-pregnancy body mass index, parity, and receipt of food assistance in pregnancy.
[c]Models adjusted for age at delivery, race, ethnicity, neighborhood deprivation index, individual-level education, Medicaid/Medicare insurance in pregnancy, pre-pregnancy body mass index, and parity.

© 2025 Chehab RF et al. *JAMA Network Open*.

eTable 4. Association of Food Insecurity in Pregnancy With Perinatal Complications Additionally Adjusting for Individual-Level Education Among 19 338 Individuals Who Responded to an Online Survey in Pregnancy at Kaiser Permanente Northern California in 2020 to 2022

Complications	Overall[a] (n= 19338)	Received food assistance in pregnancy[b] (n= 1471)	Did not receive food assistance in pregnancy[b] (n= 17867)
	Adjusted RR (95% CI)		
**Maternal**			
Gestational diabetes	1.13 (1.01-1.29)	0.85 (0.62-1.16)	1.20 (1.04-1.37)
Gestational hypertension	0.95 (0.84-1.07)	1.00 (0.73-1.38)	0.94 (0.82-1.07)
Pre-eclampsia	1.28 (1.11-1.49)	1.64 (1.06-2.53)	1.24 (1.06-1.46)
Cesarean delivery	1.07 (1.00-1.15)	1.02 (0.85-1.22)	1.08 (1.00-1.17)
**Neonatal**			
Preterm birth	1.19 (1.02-1.38)	1.00 (0.70-1.43)	1.23 (1.05-1.46)
NICU admission	1.23 (1.07-1.42)	0.93 (0.67-1.29)	1.31 (1.12-1.52)
Small-for-gestational age	1.03 (0.91-1.16)	0.76 (0.57-1.01)	1.09 (0.95-1.24)
Large-for-gestational age	0.98 (0.86-1.12)	0.88 (0.68-1.13)	1.00 (0.86-1.17)
**Composite**			
Adverse perinatal outcome	1.07 (1.02-1.13)	0.88 (0.77-1.00)	1.12 (1.06-1.18)

CI, confidence interval; NICU, neonatal intensive care unit; RR, relative risk.
Adverse perinatal outcome is a composite outcome of gestational diabetes, gestational hypertension, pre-eclampsia, preterm birth, NICU admission, and small-for-gestational age infant.
Individuals without food insecurity served as the reference category.
[a]Models adjusted for age at delivery, race, ethnicity, neighborhood deprivation index, individual-level education, Medicaid/Medicare insurance in pregnancy, pre-pregnancy body mass index, parity, and receipt of food assistance in pregnancy.
[b]Models adjusted for age at delivery, race, ethnicity, neighborhood deprivation index, individual-level education, Medicaid/Medicare insurance in pregnancy, pre-pregnancy body mass index, and parity.

© 2025 Chehab RF et al. *JAMA Network Open*.

eTable 5. Association of Food Insecurity in Pregnancy With Perinatal Complications Among 15 070 Individuals Who Responded to an Online Survey in Pregnancy at Kaiser Permanente Northern California in 2020 to 2022

Complications	Overall[a] (n= 15070)	Received food assistance in pregnancy[b] (n= 1066)	Did not receive food assistance in pregnancy[b] (n= 14004)
	Adjusted RR (95% CI)		
**Maternal**			
Gestational diabetes	1.13 (0.97-1.31)	0.85 (0.58-1.23)	1.19 (1.01-1.40)
Gestational hypertension	0.99 (0.85-1.14)	0.89 (0.62-1.27)	1.01 (0.86-1.17)
Pre-eclampsia	1.34 (1.12-1.60)	1.41 (0.84-2.37)	1.34 (1.11-1.61)
Cesarean delivery	1.07 (0.99-1.17)	1.06 (0.85-1.32)	1.07 (0.98-1.18)
**Neonatal**			
Preterm birth	1.19 (1.00-1.43)	0.87 (0.57-1.33)	1.29 (1.07-1.56)
NICU admission	1.18 (1.00-1.40)	0.82 (0.56-1.20)	1.29 (1.08-1.54)
Small-for-gestational age	1.02 (0.88-1.18)	0.72 (0.50-1.03)	1.09 (0.93-1.27)
Large-for-gestational age	1.02 (0.87-1.19)	0.87 (0.64-1.18)	1.06 (0.87-1.27)
**Composite**			
Adverse perinatal outcome	1.09 (1.02-1.15)	0.80 (0.68-0.94)	1.15 (1.08-1.23)

CI, confidence interval; NICU, neonatal intensive care unit; RR, relative risk.
Adverse perinatal outcome is a composite outcome of gestational diabetes, gestational hypertension, pre-eclampsia, preterm birth, NICU admission, and small-for-gestational age infant.
Individuals without food insecurity served as the reference category.
[a]Models adjusted for age at delivery, race, ethnicity, neighborhood deprivation index, Medicaid/Medicare insurance in pregnancy, pre-pregnancy body mass index, parity, and receipt of food assistance in pregnancy.
[b]Models adjusted for age at delivery, race, ethnicity, neighborhood deprivation index, Medicaid/Medicare insurance in pregnancy, pre-pregnancy body mass index, and parity.

© 2025 Chehab RF et al. *JAMA Network Open*.

eTable 6. Association of Food Insecurity in Pregnancy With Perinatal Complications Among 19 112 Individuals Who Responded to an Online Survey at Kaiser Permanente Northern California in 2020 to 2022 Excluding Those Who Reported Receiving Food Assistance Only From Other Sources

**Perinatal complication**	**Overall[a] (n= 19112)**	**Received food assistance in pregnancy[b] (n= 1245)**	**Did not receive food assistance in pregnancy[b] (n= 17867)**
		**Adjusted RR (95% CI)**	
**Maternal**			
Gestational diabetes	1.15 (1.01-1.31)	0.78 (0.56-1.08)	1.23 (1.07-1.40)
Gestational hypertension	0.95 (0.84-1.07)	0.98 (0.70-1.37)	0.94 (0.82-1.08)
Pre-eclampsia	1.28 (1.11-1.49)	1.54 (0.98-2.41)	1.26 (1.07-1.47)
Cesarean delivery	1.08 (1.00-1.16)	1.00 (0.82-1.22)	1.09 (1.01-1.18)
**Neonatal**			
Preterm birth	1.19 (1.01-1.39)	0.85 (0.58-1.25)	1.27 (1.07-1.49)
NICU admission	1.24 (1.07-1.42)	0.80 (0.56-1.14)	1.33 (1.15-1.54)
Small-for-gestational age	1.02 (0.90-1.15)	0.73 (0.53-1.00)	1.08 (0.95-1.22)
Large-for-gestational age	0.98 (0.85-1.12)	0.88 (0.66-1.16)	0.99 (0.85-1.16)
**Composite**			
Adverse perinatal outcome	1.08 (1.02-1.13)	0.82 (0.71-0.95)	1.13 (1.06-1.19)

CI, confidence interval; NICU, neonatal intensive care unit; RR, relative risk.
Adverse perinatal outcome is a composite outcome of gestational diabetes, gestational hypertension, pre-eclampsia, preterm birth, NICU admission, and small-for-gestational age infant.
Individuals without food insecurity served as the reference category.
[a]Models adjusted for age at delivery, race, ethnicity, neighborhood deprivation index, Medicaid/Medicare insurance in pregnancy, pre-pregnancy body mass index, parity, and receipt of food assistance in pregnancy.
[b]Models adjusted for age at delivery, race, ethnicity, neighborhood deprivation index, Medicaid/Medicare insurance in pregnancy, pre-pregnancy body mass index, and parity.

© 2025 Chehab RF et al. *JAMA Network Open*.

e**References**

1. Avalos LA, Nance N, Badon SE, Young-Wolff K, Ames J, Zhu Y, Hedderson MM, Ferrara A, Zerbo O, Greenberg M, Croen LA. Associations of COVID-19-Related Health, Healthcare and Economic Factors With Prenatal Depression and Anxiety. *Int J Public Health*. 2022;67:1604433. doi:10.3389/ijph.2022.1604433

© 2025 Chehab RF et al. *JAMA Network Open*.
