# Supplementary material for: Food Insecurity in Pregnancy, Receipt of Food Assistance, and Perinatal Complications
Source: JAMA Netw Open. 2025 Jan 23;8(1):e2455955. doi: 10.1001/jamanetworkopen.2024.55955 (PMC11758595; doi:10.1001/jamanetworkopen.2024.55955)
Supplement: Supplement 2. — Data Sharing Statement [file jamanetwopen-e2455955-s002.pdf]

Data Sharing Statement

Data

Data available: Yes
Data types: Deidentified participant data
How to access data: Please send data requests to Rana.Chehab@kp.org and Yeyi.Zhu@kp.org.
When available: With publication

Supporting Documents

Document types: None

Additional Information

Who can access the data: Researchers whose proposed use of the data has been approved.
Types of analyses: For a specified purpose.
Mechanisms of data availability: After approval of a proposal and with a signed data access agreement
Any additional restrictions: None.
